# HN1 contributes to migration, invasion, and tumorigenesis of breast cancer by enhancing MYC activity

**DOI:** 10.1186/s12943-017-0656-1

**Published:** 2017-05-11

**Authors:** Chen Zhang, Bingfei Xu, Shi Lu, Ying Zhao, Pian Liu

**Affiliations:** 1 Department of Hepatobiliary Surgery, Union Hospital, Tongji Medical College, Huazhong University of Science and Technology, Wuhan,, People’s Republic of China; 2 Cancer Center, Union Hospital, Tongji Medical College, Huazhong University of Science and Technology, Wuhan, People’s Republic of China; 3 Department of Obsterics and Gynecology, Union Hospital, Tongji Medical College, Huazhong University of Science and Technology, Wuhan, People’s Republic of China; 4 Department of pharmacy, Union Hospital, Tongji Medical College, Huazhong University of Science and Technology, Wuhan, People’s Republic of China

**Keywords:** Breast cancer, Cancer stem cell, HN1, MYC

## Abstract

**Background:**

Hematological and neurological expressed 1 (HN1) is upregulated in many tumors, but the role of HN1 in breast cancer progression and its regulatory mechanism have not been well understood.

**Methods:**

To study the role of HN1 in the initiation and progression of breast cancer, we examined HN1 levels in breast cancer cells and tissues and analyzed the relationship between HN1 levels and patient survival. We used mammosphere formation assay, side population analysis, wound healing assay, transwell assay, soft agar formation assay, and xenografted tumor model to determine the effect of HN1 on the expansion of breast cancer stem cells, and the migration, invasion and tumorigenesis of breast cancer. To determine whether HN1 regulates MYC, we used quantitative real-time PCR and Western blot analysis to assess the expression of MYC and their targeted genes to determine the phenotype caused by knockdown of MYC in breast cancer cell with HN1 overexpression.

**Results:**

In this study, we found that HN1 was upregulated in breast cancer tissues. Patients with high levels of HN1 expression had significantly shorter survival than those with low HN1 expression. In breast cancer cell line, ectopic overexpression of HN1 not only promoted the expansion of breast cancer stem cells, but also promoted cell migration, invasion, and tumorigenesis, while knockdown of HN1 reduced these effects. Furthermore, there was a positive correlation between MYC (also known as c-MYC) level and HN1 level, mechanism analysis suggested HN1 promoted the expression of MYC and its targeted genes like CDK4, CCND1, p21, CAV1, and SFRP1. Downregulation of MYC abrogated the effect of HN1 overexpression in breast cancer cell lines.

**Conclusion:**

Taken together, these data reveal that HN1 promotes the progression of breast cancer by upregulating MYC expression, and might be a therapeutic target for breast cancer.

**Electronic supplementary material:**

The online version of this article (doi:10.1186/s12943-017-0656-1) contains supplementary material, which is available to authorized users.

## Background

Breast cancer is the most frequently diagnosed malignancy and the leading cause of cancer deaths among women in worldwide. Due to the development of early diagnosis technology, the mortality rate of breast cancer is decreasing in some countries, but in other counties the mortality rate still is increasing [1]. Tumor relapse is the main cause of the high mortality of various cancers. Cancer stem cells (CSCs) are considered to be the main reason for tumor relapse and metastasis, CSCs can maintain the number or generate more CSCs by self-renewal, and generate non-CSCs progeny by differentiation. For some kinds of tumors, a CSC can initiate tumorigenesis in immunodeficient mice [2, 3]. According to the CSCs model, conventional cancer therapy only kills non-CSCs and leaves rare CSCs, but these CSCs can self-renew and differentiate to generate CSCs and non-CSCs, causing relapse, if combining antagonism CSCs drug with conventional cancer therapy methods could kill all tumor cells [4]. Breast cancer stem cells (BCSCs) were discovered in 2003 [5], several methods have been developed to study or separate BCSCs, for example, CD24^−^CD44^+^ assay [5], ALDEFLUOR assay [6], mammosphere formation assay [7], and side population (SP) assay [8].

HN1 is located on chromosome 17q25.2 and encodes a 16.5 kDa protein [9]. It has been demonstrated to upregulate in many cancers, such as lung adenocarcinoma and pancreatic carcinoma [10]. It is also a marker of ovarian cancer [11]. It is only expressed in high-grade gliomas, and HN1 knockdown inhibits tumor growth in vivo but not cell proliferation in vitro [12]. In prostate cancer, HN1 interacts with GSK3β/β-catenin destruction complex. Overexpression of HN1 promotes β-catenin degradation and negatively influences the β-catenin/E-cadherin interaction. Colony formation ability and migration ability are increased [13]. Recently, Zhanguo Zhang and colleagues find miR-132 can inhibit cell proliferation, invasion, migration and metastasis of breast cancer by targeting HN1 [14], but the role of HN1 in breast cancer and its regulatory mechanism have not been well documented. Here, we found HN1 to be upregulated in breast cancer tissues, and patients with high HN1 expression had poor prognosis. Overexpression of HN1 not only promoted the self-renewal of breast cancer stem cells, but also promoted migration, invasion, and tumorigenesis. Further analysis suggested HN1 expression had a positive correlation with MYC expression. Knockdown of MYC in HN1 overexpressing cells abrogated the phenotypes caused by HN1 overexpression, suggesting MYC is the downstream target of HN1.

## Methods

### Cells, vector, oligonucleotides, infection, and transfection

Breast cancer cells MCF-7 and T47D were obtained from the American Type Culture Collection (ATCC). They were grown in DMEM medium supplemented 10% FBS.

The coding sequence of HN1 was obtained by direct PCR of normal breast cancer cell MCF-10A cDNA and cloned into the pSin-EF2-Pur lentiviral vector. Empty vector served as negative control (Vector), the pSin-HN1-Pur (HN1) plasmid or empty vector and 2 helper plasmids pM2.G and psPAX2 were cotransfected into 293FT cells to produce lentiviruses. Lentiviruses were used to infect MCF-7 and T47D for 24 h, 5 μg/ml puromycin (Sigma) was used to construct stable HN1-overexpressing cells.

For knockdown of HN1 and MYC, small inference RNAs (siRNAs) for HN1 and MYC and their scramble control siRNAs were purchased from Ribobio (Ribobio, Inc.) were transfected at a final concentration of 10 nm using Liopofectamine 2000 (Invitrogen) according to the manufacturer’s instructions. siRNAs for HN1 which transfected into MCF-7 cells for xenografted tumor model have been modified with oMe to improve their stability.

### Tissue samples

Fresh human tissue samples including 12 breast cancer tissues and 4 normal mammary tissues were collected from Tongji Medical College, Huazhong University of Science and Technology, and were frozen snappily and stored at liquid nitrogen until use.

A cohort of 232 paraffin-embedded, archived breast cancer specimens was used to determine the clinical significance HN1, these specimens were clinically diagnosed as breast cancer at from 2000–2008. The detail information was shown in Additional file 1: Table S1. We obtained the patient’s prior written informed consent and approval from the Institutional Research Ethic Committee of Tongji Medical College, Huazhong University of Science and Technology for the use of these specimens for research purposes.

### RNA extraction and quantitative real-time PCR

Total RNA of breast cancer cells and tissues was isolated using TRIzol Reagent (Invitrogen), and used for the first strand cDNA synthesis with TransScript Reverse Transcriptase (TransGen Biotech). Quantitation of all gene transcripts was performed by quantitative real-time PCR using FastFire qPCR PreMix (SYBR Green) (TransGen Biotech) and a CFX-96 Touch Real-Time PCR Detection System (BioRad). GAPDH was used as the internal control. The primers used were as follows: HN1: forward, 5′-ATAGCTCCCGAGTTTTGCG-3′ and reverse, 5′-TTGGCCCAAGAAGCTTGA-3′; CDK4: forward, 5′-AGGCTTTTGAGCATCCCA-3′ and reverse, 5′-TCCTTAGTCGTTTCGGCT-3′; CCND1: forward 5′-TCCTCTCCAAAATGCCAGAG-3′ and reverse 5′-GGCGGATTGGAAATGAACTT-3′; p21: forward 5′-CGATGCCAACCTCCTCAACGA-3′ and reverse 5′-TCGCAGACCTCCAGCATCCA-3′, GAPDH: forward 5′GGTGGTCTCCTCTGACTTC3′ and reverse 5′CTCTTCCTCTTGTGCTCTTG-3′; CAV1: forward 5’AGATTCAGTGCATCAGCCG3’ and reverse 5′-TCTGCAAGTTGATGCGGA-3′; and SFRP1: forward 5′-TGAAGAATGGGGCTGACTG3′ and reverse 5′-TGGGGCACTCATGGTTTT-3′.

### Western blot

Human specimens and breast cancer cells were lysed with RIPA lysis Buffer (Beyotime) supplemented with cocktail protease inhibitor (Roche); protein lysates were separated by 12% SDS-PAGE and transferred to the PVDF membranes. Then, the membranes were blocked with 5% non-fat milk, and incubated with primary antibodies overnight at 4 °C, the membranes were washed in TBST 3 times for 5 min each. The membranes were incubated with HRP-conjugated secondary antibodies for 2 h at room temperature and washed 3 times with TBST for 5 min each. The bands were visualized with BeyoECL Plus reagent (Beyotime). The membranes were stripped and re-probed with an anti-β-actin antibody (HC201-02, TransGen Biotech) or GAPDH (AF0006, Beyotime) as the loading control. Primary antibodies against HN1 (HPA059729, Sigma), CDK4 (AC251, Beyotime), Cyclin D1 (AC853, Beyotime), p21 (AP021, Beyotime) and MYC (c3956, Sigma) were used.

### Immunohistochemistry (IHC) staining

IHC staining was used to assess HN1 expression in a cohort of 232 specimens, and performed according to the previous report [15]. Anti-HN1 antibody (1:200, HPA059729, Sigma) was used, and 2 investigators which blinded to the outcome scored the results of the staining independently. The xenograft tumors were fixed with formalin and embedded in paraffin for IHC staining.

### Cell migration and invasion assays

A wound healing assay was performed to determine the effect of HN1 on cell migration and performed according the previous report [14]. A Transwell chamber with an 8 μm pore with pre-coated Matrigel membrane filter (Corning) was used to determine the effect of HN1 on invasion, invasion assay was carried out as described previously [14].

### Mammosphere formation assay

For the mammosphere formation assay, breast cancer cells T47D and MCF-7 were trypsinized and resuspended in the MammoCult Basal Medium supplemented with MammoCult Proliferation Supplement (StemCell Technologies), heparin, and hydrocortisone. Then 200 cells per well were plated in ultralow-attachment 24-well plates (Corning) in triplicate. After 7 days of cell plating, the mammospheres were counted and photographed under phase contrast.

### Side population (SP) analysis

Side population analysis was performed according to the previous report [16]. Cell analysis using FACSCalibur cytometer (Becton Dickinson).

### 3’UTR luciferase reporter assay

The MYC promoter was amplified using human DNA as a template and cloned into the pGL3-Basic (Promega) vector. The primers were shown as follows: forward, 5′-GGGGTACC ATTTCTGAAGAGGACTTGTTGC-3′; reverse, 5′-GAAGATCT TTCAACAGAGGAAAACTCTTGC-3′. The vector was transfected into MCF-7 and T47D using Lipofectamine 2000. The luciferse reporter assay was carried out using Dual-Luciferase® Reporter Assay System (Promega) according to the manufacturer’s instructions.

#### Xenografted tumor model

Animal experiments were approved by the Animal Care Committee of the Tongji Medical College, Huazhong University of Science and Technology. Samples with different numbers of MCF-7 breast cancer cells (1 × 10^5^, 1 × 10^4^, and 1 × 10^3^) with HN1 overexpression or knockdown in 100 μl PBS mixed with Matrigel (1:1, BD Biosciences) were injected subcutaneously into 6-week-old female BALB/c nude mice. The mice were treated with estradiol to accelerate tumor growth. The tumor volumes were measured once every 6 days and calculated as tumor volume = Length × Width^2^/2. All mice were killed at day 60, tumors were collected and photographed.

#### Statistical analysis

Statistical analysis was performed using the SPSS 19.0 statistical software package (SPSS Inc.). All experiments were carried out at least 3 times, and the results are presented as the mean ± standard deviation. The Student’s *t* test (2-tailed) was used to statistically analyze the significance of individual groups. Survival curves were plotted using the Kaplan-Meier method and compared using the log-rank test. We defined high expression level as above the median and low expression as expression below the median. We defined patients whose survive more than 5 years as good prognosis, patients whose survive less than 5 years as poor prognosis. *P* values of <0.05 was considered statistically significant. Gene Set Enrichment Analysis (GSEA) was carried out using the Java desktop software (http://software.broadinstitute.org/gsea/index.jsp) [17].

## Results

### HN1 is upregulated in primary breast cancer tissues, and high HN1 expression correlates with poor outcome

**Fig. 1:**
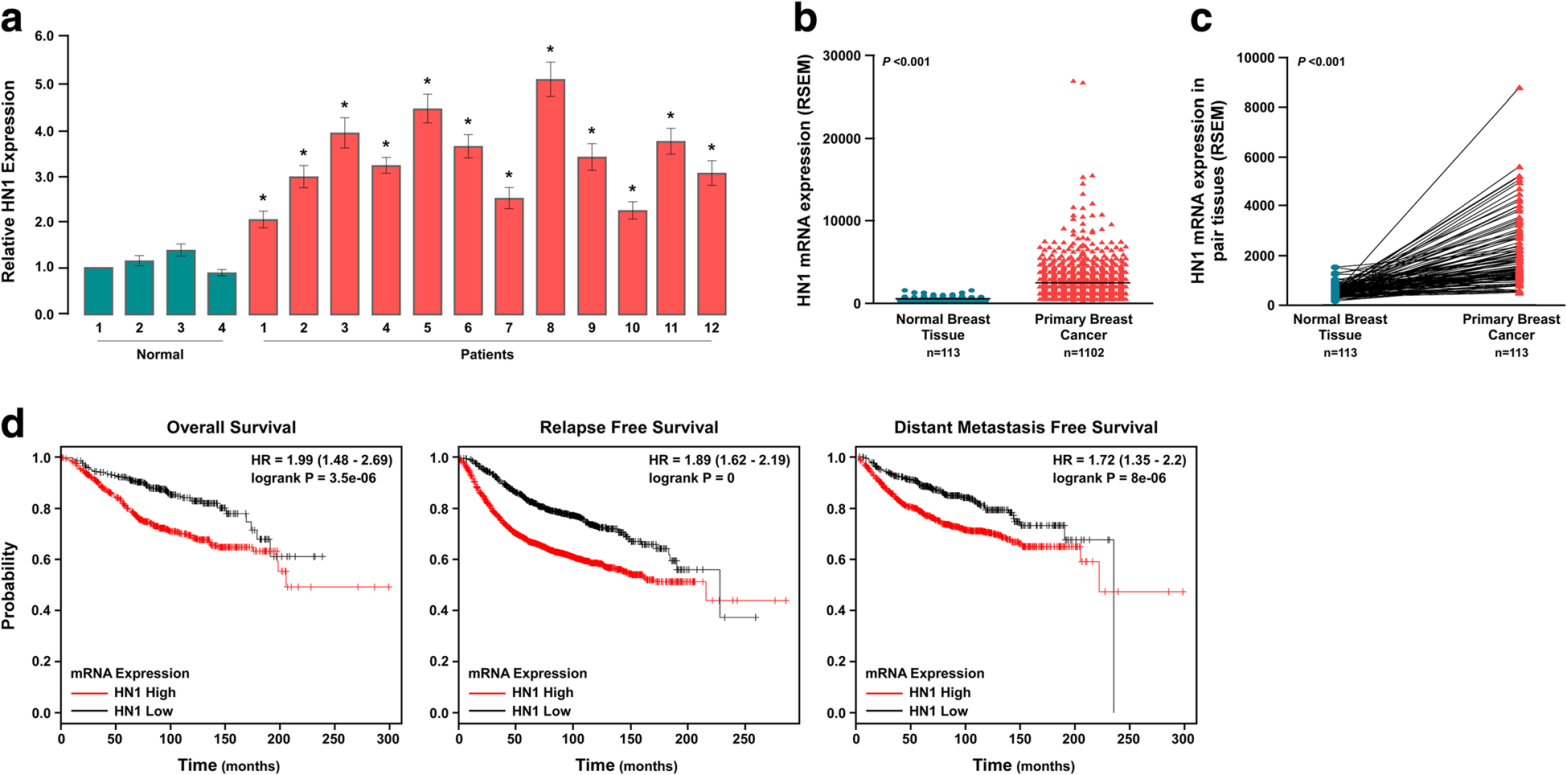
HN1 is upregulated in breast cancer tissues and correlated with poor prognosis according to the data from TCGA database. **a** *Dot plots* represent HN1 expression levels in the primary breast cancer tissues (*n* = 1102) and normal breast tissues (*n* = 113) (**P <0.001). **b** HN1 expression levels in primary breast cancer tissues (*n* = 113) and matched adjacent normal breast tissues (**P <0.001). **c** Kaplan-Meier plots with log rank test for the overall survival (*left*), relapse free survival (*middle*) and distant metastasis free survival (*right*) of breast cancer patients with high HN1 expression and low HN1 expression, respectively (**P <0.001). **d** Quantitative real-time PCR analyzed HN1 expression in 12 breast cancer tissues and 4 normal breast tissues (*P <0.05)

To investigate the role of HN1 in the development and progression of breast cancer, patient survival and gene expression data for 1102 breast cancers were downloaded from the Cancer Genome Atlas (TCGA) database. We first analyzed HN1 expression in 1102 primary breast cancer tissues and 113 normal breast tissues and found HN1 to be significantly upregulated in primary breast cancer tissues (Fig. 1a, *P* <0.001). We confirmed this result using 113 primary breast cancer tissues and their matched adjacent normal breast tissues and found that HN1 was also statistically significantly upregulated in primary breast cancer tissues (Fig. 1b, *P* <0.001).

Kaplan-Meier survival curves suggested that the overall survival of the patients with high levels of HN1 expression was significantly longer than those with low levels of HN1 expression (Fig. 1C, *P* = 3.5e-06), it was consistent with the result analyzed using BreastMark database by Zhang and colleagues [14]. Patients with high levels of HN1 expression had a significantly shorter overall survival compared to those with low HN1 expression in those without relapse (*P* = 0), and in those without distant metastasis (Fig. 1c, *P* = 8e-06).

**Fig. 2:**
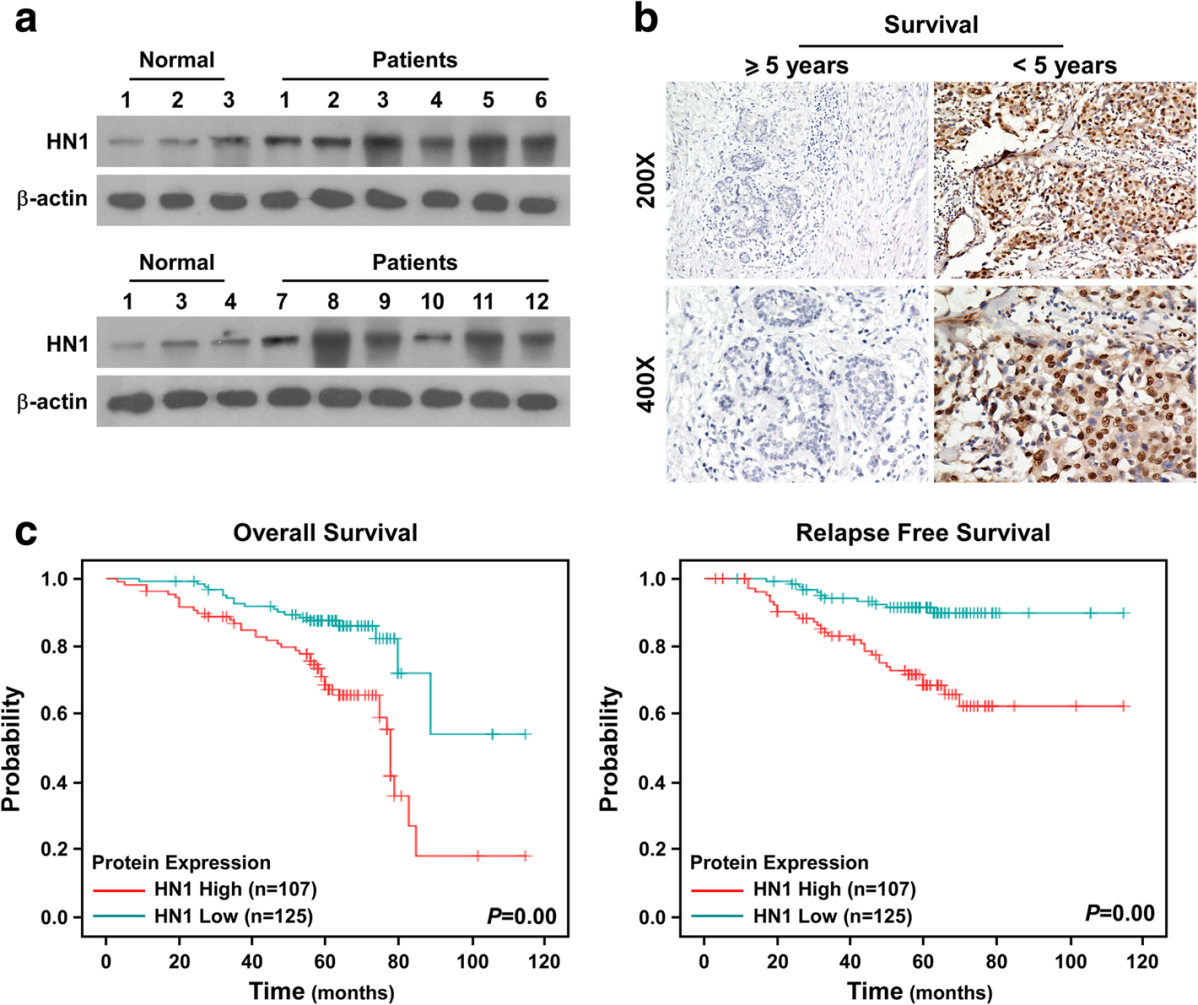
HN1 is upregulated in breast cancer tissues and correlates with poor prognosis according to clinical specimens. **a** *Western blot* assay of HN1 expression in normal breast tissues and breast cancer tissues. **b** IHC staining analysis of HN1 expression in patients with good prognosis and patients with poor prognosis. **c** Kaplan-Meier plots with log rank test for overall survival and relapse-free survival of breast cancer patients with high HN1 expression and low HN1 expression, respectively. Each bar represents mean ± SD of 3 independent experiments

According to the data of TGCA database, HN1 was found to be upregulated in primary breast cancer tissues. We further confirmed this argument using 16 specimens, including 4 normal mammary tissues and 12 breast cancer tissues. Quantitative real-time PCR assay suggested HN1 was upregulated in breast cancer tissues compared to normal breast tissues (Fig. 1d). Western blot assay found HN1 levels were higher in primary breast cancer tissues than that of in normal breast tissues (Fig. 2a). We used IHC to assess HN1 expression in a cohort of 232 breast cancer patients, and found HN1 located in cytoplasm and nucleus. Patients with good prognosis had low HN1 expression, and patients with poor prognosis had high levels of HN1 expression (Fig. 2b). Kaplan-Meier survival curves suggested both overall survival and relapse-free survival of the patients with high HN1 expression were significantly longer than those with low levels of HN1 expression (Fig. 2c, *P* = 0.00). These results were consistent with results from TCGA database. Taken together, these findings suggested HN1 was upregulated in breast cancer tissues, patients with high HN1 expression had poor outcome.

### HN1 overexpression promoted self-renewal of breast cancer stem cells (BCSCs)

**Fig. 3:**
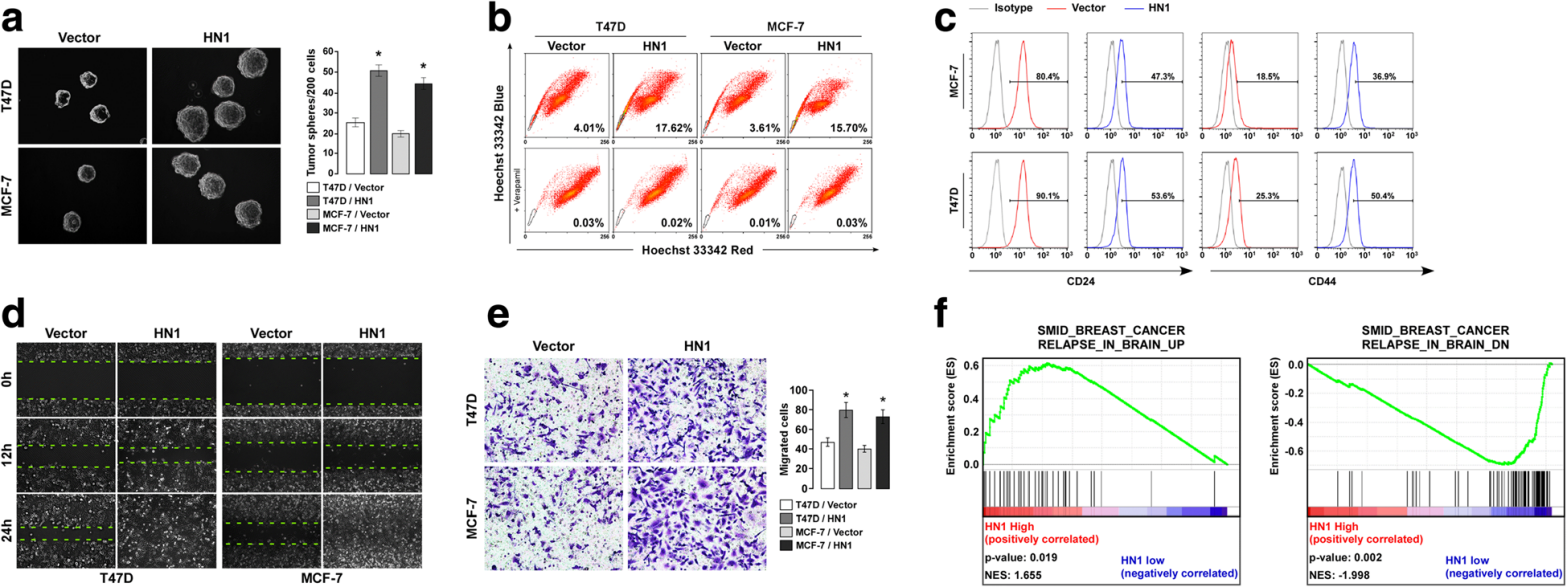
HN1 promotes the self-renewal of BCSCs and migration and invasion of breast cancer cells. **a** Mammosphere formation analysis for the self-renewal ability of BCSCs with HN1 overexpression (*P <0.05). **b** SP analysis for the self-renewal ability of BCSCs with HN1 overexpression. **c** Flow cytometry analysis of the relative size of the CD24^−^CD44^+^ population after HN1 overexpression. **d** Wound healing assay showing the migration ability of MCF-7 and T47D with HN1 overexpression. **e** Transwell invasion analysis showing the invasion ability of indicated cells with HN1 overexpression (*P < 0.05). **f** Correlation of HN1 mRNA levels and the gene signatures about breast cancer relapse in brain using GSEA (*P <0.05). Each bar represents mean ± SD of 3 independent experiments

Cancer stem cells (CSCs) play critical role in tumor relapse and metastasis. Considering the fact of poor prognosis for patients with higher levels of HN1 expression, we were wondering if there was any relationship between HN1 and BCSCs. Mammosphere formation assay and Hoechst negative side population (SP) cells analysis were used to determine the role of HN1 in BCSCs, the results demonstrated that overexpression of HN1 was significantly closely associated with the formation of more and bigger mammosphere (Fig. 3a), and the increased percentage of SP cells (Fig. 3b). CD24^−^CD44^+^ is a marker for BCSCs, HN1 overexpression increased the percentage of CD24^−^CD44^+^ population. These suggested HN1 overexpression promoted self-renewal of BCSCs. We also knocked down HN1 in the same cells, mammosphere formation assay suggested that HN1 knockdown significantly reduced the number and volume of mammosphere (Additional file 2: Figure S1A). SP analysis suggested that HN1 knockdown reduced the percentage of SP cells (Additional file 2: Figure S1B). These results revealed that HN1 promoted the self-renewal of BCSCs.

### HN1 overexpression promotes breast cancer migration, invasion, and tumorigenesis

To determine the role of HN1 in breast cancer, we used wound healing assay and transwell analysis to determine the effect of HN1 on cell migration and invasion, and found that HN1 overexpression promoted cell migration and invasion of MCF-7 and T47D breast cancer cells (Fig. 3c and d). HN1 knockdown inhibited cell migration and invasion of MCF-7 and T47D (Additional file 2: Figure S1C and D). The brain is one of main sites of breast cancer metastasis, and patients with brain metastasis have poor prognosis. The median survival of untreated patients is about 1 month [18]. GSEA analysis showed HN1 expression to be positively correlated with genes overexpressed in breast cancer relapse in the brain. HN1 expression was found to be negatively correlated with genes downregulated in breast cancer relapse in brain (Fig. 3e).

**Fig. 4:**
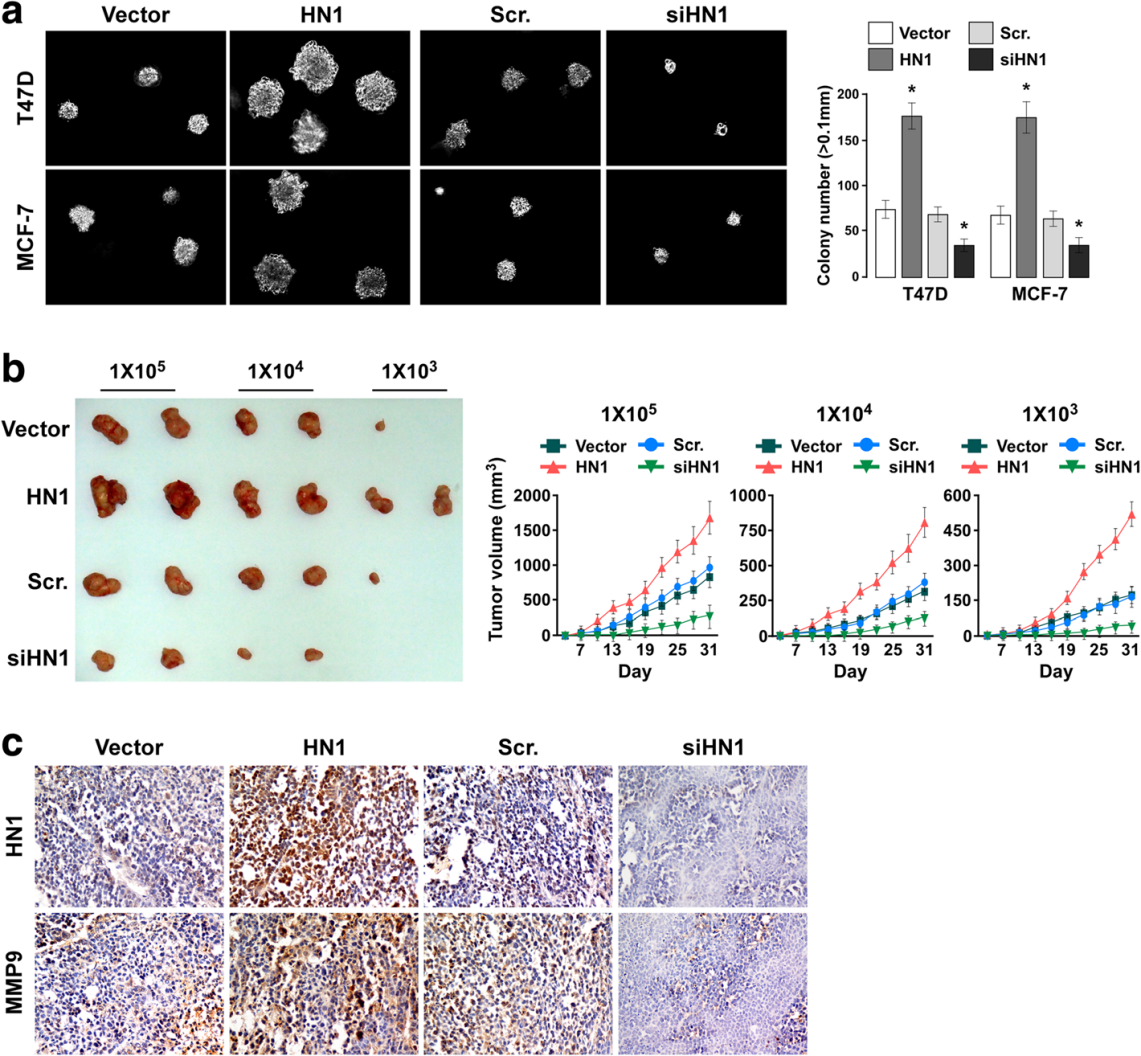
HN1 promotes tumorigenesis of breast cancer cells in vitro and in vivo. **a** Soft agar growth assay of tumorigenesis in vitro in indicated cells with HN1 overexpression or knockdown (*P <0.05). **b** Xenograft model in nude mice (*left*) and tumor volume growth curves (*right*). **c** IHC staining of MMP9 in xenograft tumors. Each bar represents mean ± SD of 3 independent experiments

We used soft agar growth assay to investigate the effect of HN1 on tumorigenesis, and found overexpression of HN1 in indicated cells significantly promoted tumorigenesis in vitro (Fig. 4a). HN1 knockdown significantly inhibited tumorigenesis in vitro (Fig. 4a). A tumorigenesis assay in female nude mice was used to confirm whether HN1 promoted tumor growth in vivo, and results showed that HN1 overexpression promoted xenograft growth, and the tumor volume was bigger when HN1 expression was higher. HN1 knockdown inhibited xenograft growth, and the tumor volume was smaller (Fig. 4b). These results suggested that HN1 overexpression promoted tumorigenesis, and knockdown of HN1 inhibited tumorigenesis. We fixed xenograft tumor using formalin and embedded them in paraffin for IHC staining. Matrix metalloproteinases (MMPs) play a central role in metastasis. For example, MMP9 has high levels of expression in breast cancer, especially in breast cancer with strong capacity for distant metastasis. It has been used as a predictive marker for breast cancer invasion and metastasis [19–21]. Overexpression of MMP9 in breast cancer has been shown to promote invasion and metastasis [22]. IHC analysis indicated that overexpression of HN1 enhanced MMP9 expression, knockdown of HN1 decreased MMP9 expression (Fig. 4c), suggesting HN1 overexpression promoted invasion and metastasis.

### HN1 activates MYC pathway

**Fig. 5:**
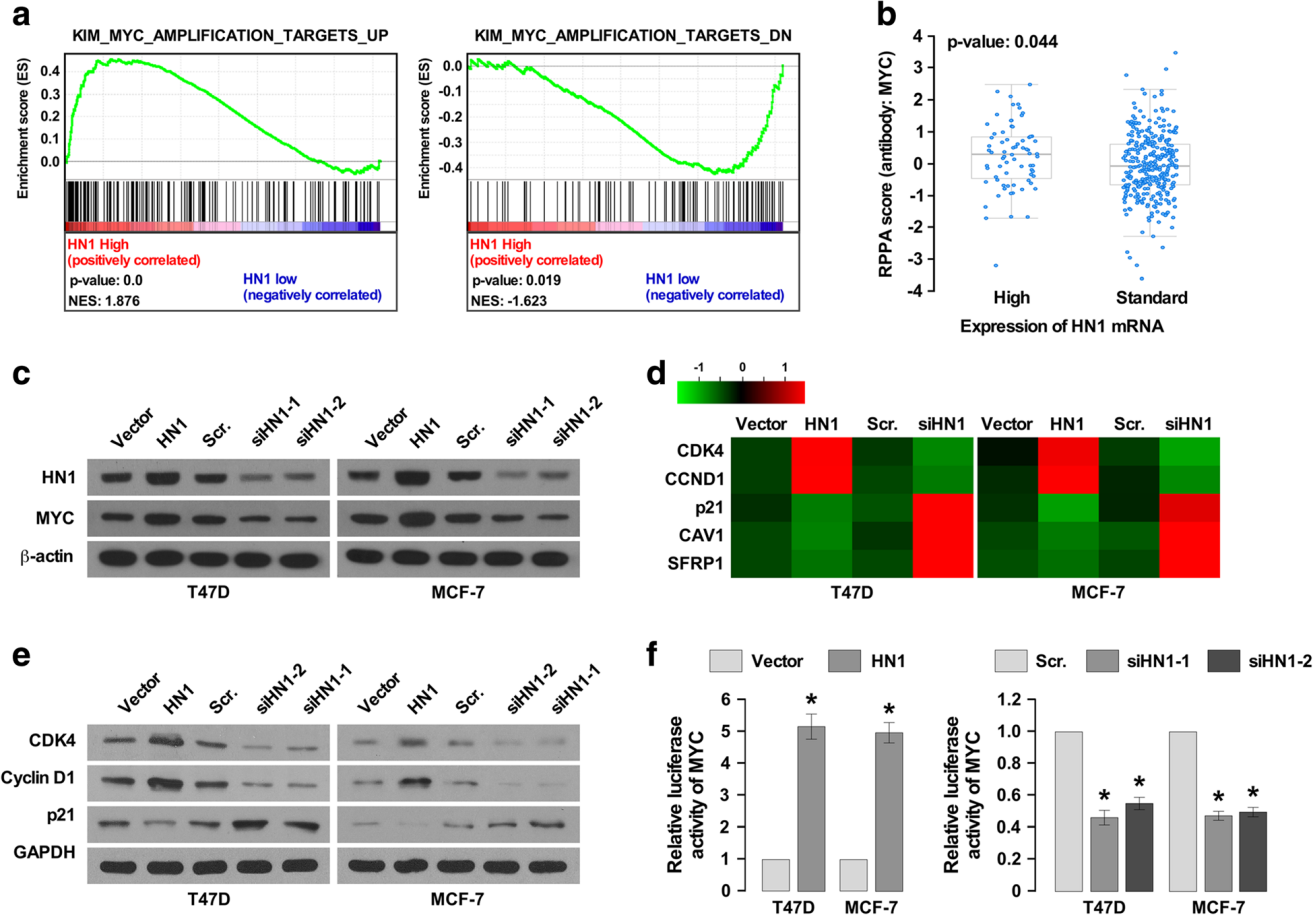
HN1 regulates MYC expression. **a** Enrichment plot, indicating a significant correlation between HN1 mRNA level and the MYC regulated gene signatures (*P <0.05). **b** Correlation of MYC and HN1 expression (*P* = 0.044). **c** Western blot analysis of the expression of HN1 and MYC in indicated cells with HN1 overexpression or knockdown, β-actin was used as the loading control. **d** The heatmap for the expression of MYC targeted genes including CDK4, CCND1, p21, CAV1, and SFRP1 after overexpression or knockdown of HN1. Quantitative real-time PCR was used to determine their expression. **e** Western blot analyzed the expression of CDK4, Cyclin D1 and p21 after HN1 overexpression or knockdown. GAPDH was used as the loading control. **f** Luciferase assay of MYC activity after overexpression or knockdown of HN1(*P <0.05). Each bar represents mean ± SD of 3 independent experiments

GESA analysis showed HN1 expression was positively correlated with MYC-activated target gene expression and negatively correlated with MYC-suppressed gene expression (Fig. 5a). The reverse-phase protein arrays (RPPA) determines protein expression in a large number of samples [23]. Quantitative protein expression profiles have been generated using RPPA, the data could be available at the TCGA database, we found HN1 expression positively correlated with MYC expression in breast cancer samples, the HN1 mRNA increased the protein level of MYC (Fig. 5b), suggesting HN1 might be a regulator of MYC, and could activate MYC pathway. We further confirmed this hypothesis in breast cancer cells MCF-7 and T47D. Western blot assay suggested overexpression of HN1 promoted MYC expression, and knockdown of HN1 inhibited MYC expression (Fig. 5c).

We also analyzed the expression of MYC targeted genes using quantitative real-time PCR and Western blot analysis. CDK4 and CCND1 were found to be transactivated by MYC [24], p21, CAV1 and SFRP1 are suppressed by MYC. HN1 knockdown was found to inhibit CDK4 and CCND1 expression and to promote p21, CAV1 and SFRP1 expression, and vice versae (Fig. 3d and e). These findings also suggested HN1 could regulate CDK4 and CCND1 to promote G1/S transition, p21 is a cell cycle inhibitor, inhibition of p21 promoted cell cycle progression. CAV1 is a tumor suppresser, and inhibition of CAV1 promotes cell proliferation and invasion [25, 26]. Wnt signaling promotes breast cancer progression and the self-renewal of BCSCs [27]. SFRP1 is a Wnt antagonist that inhibits Wnt signaling [28]. The promoter sequence of MYC was subcloned into pGL3vector, and cotransfected with HN1-overexpressing vector or siRNA for HN1. A luciferase activity assay showed HN1 overexpression enhanced the luciferase activity, inhibition of HN1 decreased the luciferase activity (Fig. 5f), suggesting HN1 indeed regulated MYC expression.

**Fig. 6:**
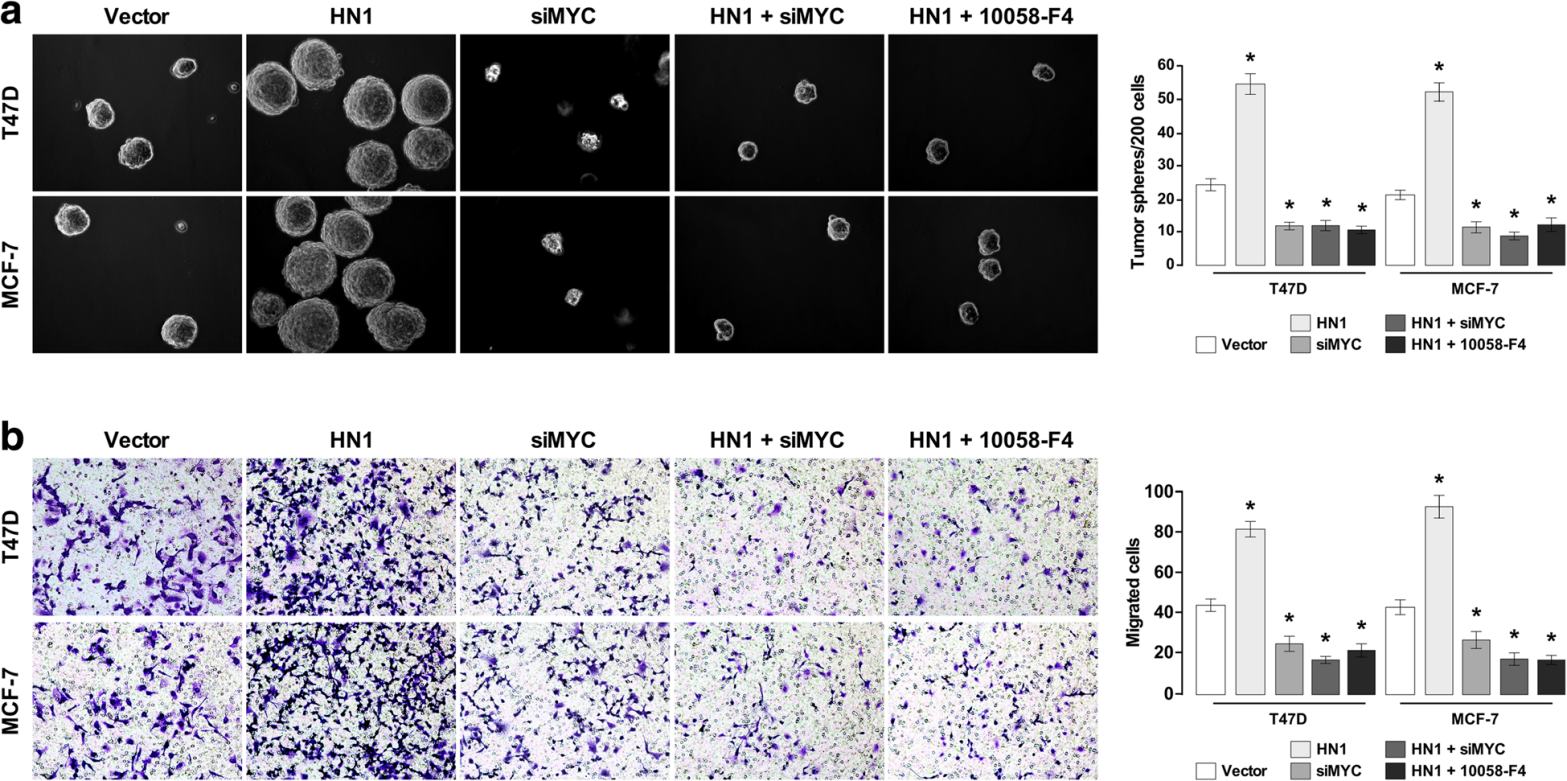
Inhibition of MYC suppresses the phenotypes caused by HN1 overexpression. **(a)** Mammosphere formation analysis of the self-renewal ability of BCSCs. MCF-7 and T47D cells overexpressing HN1 were exposed to MYC-siRNA and MYC inhibitor 10058-F4 (*P <0.05). **b** Transwell invasion analysis showed the invasion ability of indicated cells. MCF-7 and T47D cells with overexpression of HN1 were exposed to MYC-siRNA and MYC inhibitor 10058-F4 (*P <0.05). Each bar represents mean ± SD of 3 independent experiments

MYC is a famous oncogene, its knockdown significantly inhibited mammosphere formation and breast cancer cell invasion (Fig. 6a and b). We further demonstrated whether HN1 can regulate the proliferation and invasiveness of breast cancer and the self-renewal of BCSCs by regulating MYC expression. We used MYC small interference RNA (siMYC) and MYC inhibitor 10058-F4 to inhibit MYC expression [29]. A mammosphere formation assay suggested the number and size of the mammosphere formationby breast cancer cells with HN1 overexpression and inhibition of MYC were significantly reduced (Fig. 6a). A transwell assay showed that invasion was significantly inhibited in breast cancer cells that overexpressed HN1 and inhibition of MYC (Fig. 6b). Overall, HN1 promoted MYC migration, invasion, and tumorigenesis of breast cancer and the self-renewal of BCSCs through upregulating MYC expression.

## Discussion

In the present study, we used the expression profiles of normal and malignant breast tissue downloaded from TCGA database to analyze HN1 expression. Results showed HN1 to be upregulated in breast cancer tissues. Patients with high levels of HN1 expression had poor outcomes. Using clinical samples, we found the results to be similar to those gleaned from analysis of the data from TCGA database, suggesting HN1 indeed was upregulated in breast cancer tissues. We further studied the role of HN1 in migration, invasion, and tumorigenesis. We found overexpression of HN1 promoted migration, invasion and tumorigenesis. GSEA analysis suggested there was a positive correlation between HN1 expression and the expression of genes which were upregulated in breast cancer relapse in brain. IHC analysis of xenograft tumor showed HN1 overexpression enhanced MMP9 expression, and knockdown of HN1 inhibited MMP9 expression. These findings suggested that HN1 promoted breast cancer invasion and metastasis. But whether HN1 promotes metastasis in vivo will be demonstrated further.

We also studied the role of HN1 in BCSCs and found overexpression of HN1 to enhance mammosphere formation and increase the relative size of the SP cell population. The xenograft tumor model suggested that when 1 × 10^3^ tumors cells were injected into the nude mice, only half of the mice in the control group developed xenograft tumors, but all nude mice in the stable HN1 overexpression group did, and none of the nude mice in the HN1 stable knockdown group did. This suggested that overexpression of HN1 promoted the self-renewal of BCSCs, and knockdown of HN1 inhibited it. This should be confirmed using NOD/SCID mice, although nude mice are also used for CSC research [30].

MYC is a famous oncogene, and overexpresses in various tumors. It plays important roles in tumor proliferation, apoptosis and tumorigenesis [31]. We found breast cancer patients with high HN1 expression had high MYC expression, suggesting HN1 might regulate MYC expression, overexpression of HN1 increased MYC-activating genes expression, and decreased MYC-suppressing genes expression and vice versa. These suggesting HN1 was the upstream of MYC. Threonine 58 and serine 62 in the N-terminus of MYC can be phosphorylated by GSK-3β and ERK, respectively, phosphorylation of Serine 62 stabilizes MYC, phosphorylation of Threonine 58 destabilizes MYC and promotes proteasomal degradation of MYC, inhibition GSK-3β activity increases the stability of MYC [32]. HN1 might inhibit the ability of GSK-3β to increase the stability of MYC. Phosphorylation of GSK3-β on the S9 residue inhibits its kinase activity, knockdown of HN1 in prostate cancer cells PC-3 and LNCaP inhibits pGSK-3β^(S9)^ [10], we concluded that HN1 could increase the stability of MYC by suppressing the kinase activity of GSK-3β. Western blot assay suggested HN1 overexpression increased pGSK-3β^(S9)^, HN1 knockdown inhibited pGSK-3β^(S9)^ (Additional file 3: Figure S2), confirming our conference.

## Conclusions

We found HN1 to be upregulated in breast cancer tissues. Overexpression of HN1 not only promoted breast cancer migration, invasion, and tumorigenesis and the self-renewal of BCSCs. Mechanism analysis suggested MYC was the downstream of HN1. HN1 promoted the progression of breast cancer through upregulating MYC.

## Additional files

Additional file 1: Fresh human tissue samples including 12 breast cancer tissues and 4 normal mammary tissues. (XLSX 17.3 kb)

Additional file 2: Figure S1. HN1 knockdown inhibits the self-renewal of BCSCs and invasion and migration of breast cancer cells. (A). Mammosphere formation analysis for the self-renewal ability of BCSCs with HN1 knockdown (*P <0.05). (B). SP analysis for the self-renewal ability of BCSCs with HN1 knockdown. (C). Wound healing assay showing the migration ability of MCF-7 and T47D with HN1knockdown. (D) Transwell invasion analysis showing the invasion ability of indicated cells with HN1 overexpression (*P <0.05). Each bar represents mean ± SD of 3 independent experiments. (TIF 17873 kb)

Additional file 3: Figure S2. Western blot analyzed the expression of pGSK-3β^(S9)^ after HN1 overexpression or knockdown. GAPDH was used as the loading control. (TIF 141 kb)
