# Supplementary figures and images for: HN1 contributes to migration, invasion, and tumorigenesis of breast cancer by enhancing MYC activity
Source: Mol Cancer. 2017 May 11;16:90. doi: 10.1186/s12943-017-0656-1 (PMC5426009; doi:10.1186/s12943-017-0656-1)

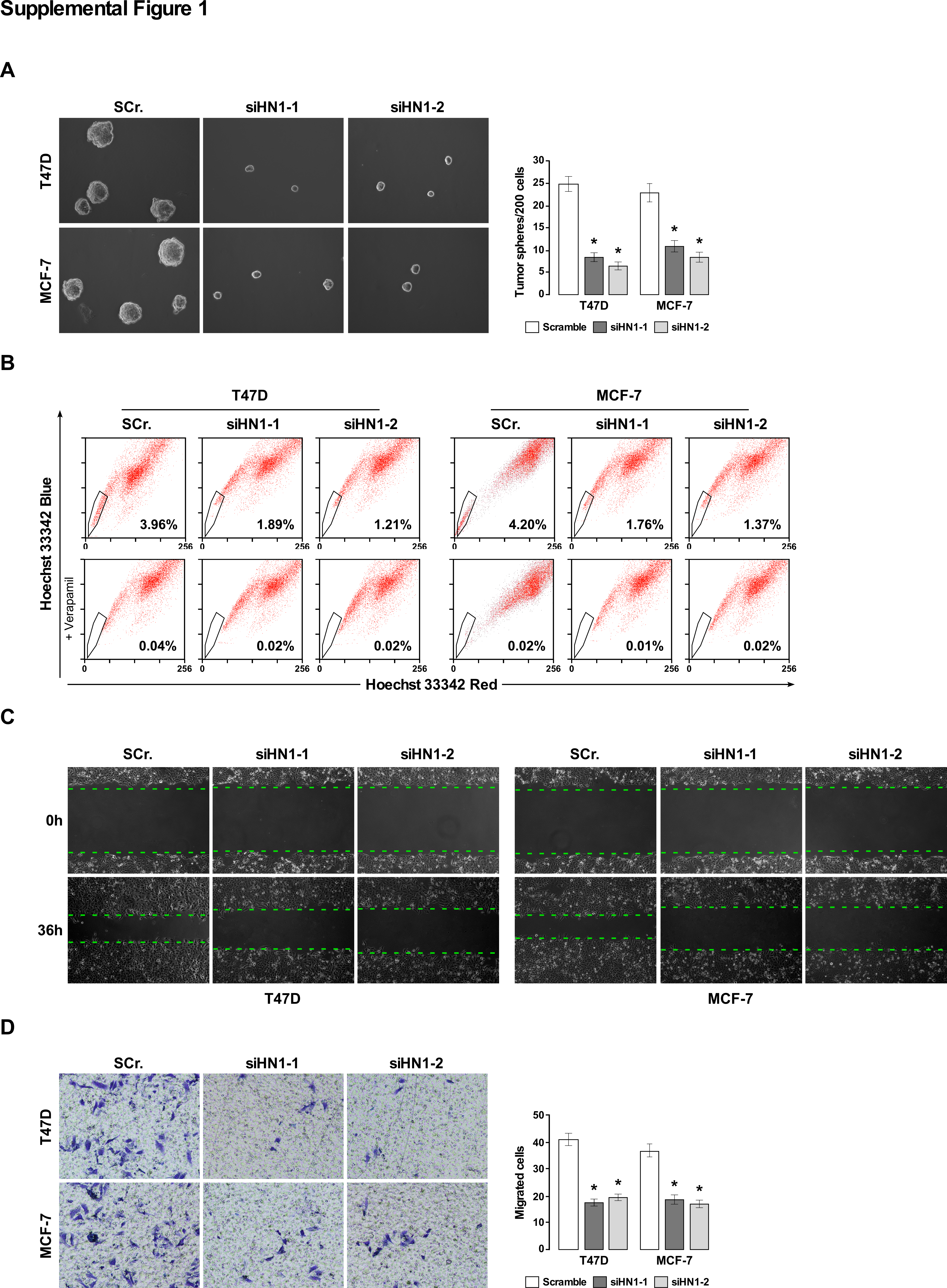

Supplement: Supplementary file 2 — HN1 knockdown inhibits the self-renewal of BCSCs and invasion and migration of breast cancer cells. (A). Mammosphere formation analysis for the self-renewal ability of BCSCs with HN1 knockdown (*P <0.05). (B). SP analysis for the self-renewal ability of BCSCs with HN1 knockdown. (C). Wound healing assay showing the migration ability of MCF-7 and T47D with HN1knockdown. (D) Transwell invasion analysis showing the invasion ability of indicated cells with HN1 overexpression (*P <0.05). Each bar represents mean ± SD of 3 independent experiments. (TIF 17873 kb) [file 12943_2017_656_MOESM2_ESM.tif]
